# Operative hysteroscopy versus vacuum aspiration for incomplete spontaneous abortion (HY-PER): study protocol for a randomized controlled trial

**DOI:** 10.1186/s13063-015-0900-1

**Published:** 2015-08-19

**Authors:** Cyrille Huchon, Martin Koskas, Aubert Agostini, Cherif Akladios, Souhail Alouini, Estelle Bauville, Nicolas Bourdel, Hervé Fernandez, Xavier Fritel, Olivier Graesslin, Guillaume Legendre, Jean-Philippe Lucot, Isabelle Matheron, Pierre Panel, Cyril Raiffort, Arnaud Fauconnier

**Affiliations:** Department of Gynecology and Obcstetrics, CHI Poissy-St-Germain, 10 Rue du champ Gaillard, BP 3082, CEDEX 78303, Poissy, France; Equipe d’accueil EA 7285 Risques, Cliniques et Sécurité en Santé des Femmes et en Santé Périnatale, Université Versailles-Saint-Quentin en Yvelines, 78180 Montigny le Bretonneux, France; Department of Gynecology and Obstetrics, APHP, Hôpital Bichat, Paris Diderot University, Paris, France; Department of Gynecology and Obstetrics, Hôpital La Conception, 147, Boulevard Baille, Cedex 5 13385, Marseille, France; Department of Gynecology and Obstetrics, Strasbourg University Hospital, 1 Avenue Molière, 67000 Strasbourg, France; Department of Gynecologic Surgery and Obstetrics, CHR Orleans, 1 Porte Madeleine, 45000 Orléans, France; Department of Gynecology and Obstetrics, Rennes University Hospital, 16 Rue de Bulgarie, B.P. 90347, Cedex 2, F-35203 Rennes, France; Department of Gynecologic Surgery, CHU Estaing Clermont Ferrand, 63058 Cedex 1, Clermont Ferrand, France; Faculté de Medicine, ISIT – Université d’Auvergne, Place Henri Dunant, 63000 Clermont-Ferrand, France; Department of Gynecology and Obstetrics, AP-HP, Hôpital Bicêtre, 78 Rue du Général Leclerc, Le Kremlin Bicêtre, 94270 France; CESP-INSERM U1018, 82 Rue du Général Leclerc, Le Kremlin Bicêtre, 94276 France; Université Paris Sud, 63 Rue Gabriel Péri, Le Kremlin Bicêtre, 94270 France; Department of Gynecology and Obstetrics, CHU de Poitiers, Université de Poitiers, Faculté de Médecine et Pharmacie, Inserm CIC1402, 2 Rue de la Milétrie, F-86000 Poitiers, France; Department of Gynecology and Obstetrics, Hôpital Alix de Champagne, CHU de Reims, 45 Rue Cognacq-Jay, 51092 Reims, France; Department of Gynecology and Obstetrics, CHU d’Angers, 4, Rue Larrey, 49033 Cedex 01, AngersPays De La Loire, France; Department of Gynecology and Obstetrics, Hôpital Jeanne-de-Flandre, CHRU de Lille, 59037 Lille, France; Department of Gynecology and Obstetrics, Centre Hospitalier Intercommunal, Villeneuve-Saint-Georges, Paris, France; Department of Gynecology and Obstetrics, Centre Hospitalier de Versailles, 177, Rue de Versailles, 78157 Le Chesnay, France; Department of Gynecology and Obstetrics, APHP, Hôpital Louis Mourier, Département Hospitalier Universitaire Risque et Grossesse, Colombes, France Université Paris-Diderot, Paris, France; Department of Gynecology and Obcstetrics, CHI Poissy-St-Germain, 10 Rue du champ Gaillard, BP 3082, 78300 Poissy, France

**Keywords:** Incomplete miscarriage, Hysteroscopy, Vacuum aspiration, Aspirative curettage, Pregnancy loss, RCT

## Abstract

**Background:**

Incomplete spontaneous abortions are defined by the intrauterine retention of the products of conception after their incomplete or partial expulsion. This condition may be managed by expectant care, medical treatment or surgery. Vacuum aspiration is currently the standard surgical treatment in most centers. However, operative hysteroscopy has the advantage over vacuum aspiration of allowing the direct visualization of the retained conception product, facilitating its elective removal while limiting surgical complications. Inadequately powered retrospective studies reported subsequent fertility to be higher in patients treated by operative hysteroscopy than in those treated by vacuum aspiration. These data require confirmation in a randomized controlled trial comparing fertility rates between women undergoing hysteroscopy and those undergoing vacuum aspiration for incomplete spontaneous abortion.

**Methods:**

After providing written informed consent, 572 women with incomplete spontaneous abortion recruited from 15 centers across France will undergo randomization by a centralized computer system for treatment by either vacuum aspiration or operative hysteroscopy. Patients will not be informed of the type of treatment that they receive and will be cared for during their hospital stay in accordance with standard practices at each center. The patients will be monitored for pregnancy or adverse effects by a telephone conversation or questionnaire sent by e-mail or post over a period of two years. In cases of complications, failure of the intervention or diagnosis of uterine cavity disease, patient care will be left to the discretion of the medical center team.

**Discussion:**

If our hypothesis is confirmed, this study will provide evidence that the use of operative hysteroscopy can increase the number of pregnancies continuing beyond 22 weeks of gestation in the two-year period following incomplete spontaneous abortion without increasing the incidence of morbidity and peri- and postoperative complications. The standard surgical treatment of this condition would thus be modified. This study would therefore have a large effect on the surgical management of incomplete spontaneous abortion.

**Trial registration:**

ClinicalTrials.gov Identifier: NCT02201732; registered on 17 July 2014.

**Electronic supplementary material:**

The online version of this article (doi:10.1186/s13063-015-0900-1) contains supplementary material, which is available to authorized users.

## Background

Incomplete spontaneous abortions are defined as the intrauterine retention of the products of conception after non-discharge or partial discharge of the egg. After miscarriage, 60 % of women wishing to fall pregnant again are able to do so within 2 years, and 80 % after 5 years [1].

Intrauterine retention after miscarriage is treated by expectant or medical management or surgery [2, 3]. Surgical treatment may be performed immediately or following the failure of expectant or medical treatment. Vacuum aspiration is currently the standard surgical treatment in most centers. However, it is typically carried out in a blind manner, and can therefore lead to the persistence of intrauterine retention, which may not be diagnosed initially. This persistence may be complicated by chronic infection and may require further surgical intervention. The abrasion of the uterine lining that occurs during this blind procedure can also cause simple or complex intrauterine adhesions. The prevalence of these adhesions is around 20 % after curettage for spontaneous abortion [4, 6] and 40 % for repeat procedures [7]. Such adhesions, like chronic endometritis resulting from persistent retention after curettage, may be detrimental to subsequent fertility and require further surgical intervention.

Some studies have reported that hysteroscopy is a beneficial surgical treatment for intrauterine retention after miscarriage, with treated women falling pregnant again within 2 years of the initial intervention in more than 70 % of cases [7, 8]. Hysteroscopy makes it possible to visualize the retention product, its elective removal and the integrity of the cavity directly, without trauma to the adjacent endometrium, while limiting the complications of surgery and the number of repeat interventions due to retention or adhesions, as indicated in a systematic review that retrieved a low frequency of intrauterine adhesions (5.7 %) after the hysteroscopic removal of retained products of conception [9]. Furthermore, hysteroscopy may facilitate the diagnosis of abnormalities or diseases of the uterine cavity (fibroids, polyps), which may be responsible for spontaneous abortions. These abnormalities may be amenable to surgical treatment, improving the management of patients and their prognosis in terms of fertility.

Two retrospective studies have compared the effectiveness of hysteroscopy and curettage for the management of post-abortion and postpartum retention [10, 11]. The first study compared 24 women who underwent curettage with 46 women who underwent hysteroscopic resection. Overall, 20 % of women in the curettage group required secondary hysteroscopy to empty the uterus, whereas no further surgery was required in the hysteroscopy group [10]. Furthermore, the women in the hysteroscopy group fell pregnant more quickly after the intervention than those in the curettage group. The second study, based on a before/after design, compared 42 women who underwent ultrasound-guided curettage with 53 women who underwent hysteroscopy [11]; these 95 women corresponded to 88 cases of failed curettage after miscarriage and seven cases of postpartum retention of the placenta after cesarean section. This study also found that the mean time to conception was shorter among women wishing to fall pregnant again in the hysteroscopy group than in those of the curettage group. Furthermore, despite preliminary curettage in cases of trophoblastic retention, this study also reported that the rate of pregnancy was significantly higher in the hysteroscopy group than in the curettage group (69 % vs 60 %, respectively). Pooling these two studies in a meta-analysis, Smorgick et al. obtained an odds ratio of 1.7 (95 % CI 0.7–3.8, *p* = 0.1) for pregnancy in the hysteroscopy group, with the curettage group used as the reference group [9]. These results provide support for the idea that incomplete spontaneous abortions can be managed by hysteroscopy. However, the retrospective design of these studies, the heterogeneity of the patients included and the low power are such that it is not possible to determine whether hysteroscopy is the best treatment option for intrauterine retention. A randomized, controlled trial is required to evaluate the place of hysteroscopy among the various treatment options.

The main objective of our study is to compare fertility rates between women undergoing hysteroscopy and those undergoing vacuum aspiration for intrauterine retention following miscarriage, in a pragmatic approach. The secondary objectives of the study are: (1) to compare times to conception following the two surgical treatments and (2) to investigate whether hysteroscopy is associated with fewer peri- and postoperative complications and repeat procedures.

## Methods

Items from the World Health Organization Trial Registration Data Set for the Operative hysteroscopy versus vacuum aspiration for incomplete spontaneous abortion (HY-PER) trial are shown in Additional file 1. The TIDieR (Template for Intervention Description and Replication) and SPIRIT (Standard Protocol Items: Recommendations for Interventional Trials) 2013 Checklists are presented in Additional files 2 and 3.

### Study design

The HY-PER trial is a randomized, open, multicenter trial comparing vacuum aspiration with hysteroscopy in women with incomplete spontaneous abortion.

### Ethics

Protocol version 1.1 for this study, dated 06/16/2014, was approved by the “Comité de Protection des Personnes” (CPP) Ile de France XI (no. 14040) and the ANSM (no. 140516B-22). Modified protocol version 2.1 dated 5 June 2015 was approved by the CPP Ile de France XI (no. 14040) on 6 November 2015. Any further modification of the protocol will require approval from the CPP and will be communicated to the local investigators.

### Main outcome

The main outcome is intrauterine pregnancy lasting up to least 22 weeks of gestation in the 2 years after the management of incomplete spontaneous abortion.

### Secondary outcomes

The secondary outcomes are as follows: time to conception; pregnancy outcome: incidence of miscarriage and ectopic pregnancy during the two-year follow-up period; number of surgical complications according to the Clavien-Dindo classification [12]; and the incidence of secondary surgical interventions for the management of intrauterine retention after miscarriage.

### Recruitment

Patients will be recruited from 15 centers (general and university hospitals) throughout France: Centre Hospitalier Intercommunal de Poissy-St-Germain en Laye, Poissy; Hôpital Bichat (AP-HP), Paris; Hôpital La Conception (AP-HM), Marseille; Strasbourg University Hospital, Strasbourg; CHR Orléans, Orléans; Rennes University Hospital, Rennes; CHU Estaing, Clermont Ferrand; Hôpital Bicêtre (AP-HP), Le Kremlin Bicêtre; CHU de Poitiers, Poitiers; Hôpital Alix de Champagne, Reims; CHU d’Angers, Angers; Hôpital Jeanne-de-Flandre, Lille; Centre Hospitalier Intercommunal de Villeneuve-Saint-Georges, Villeneuve-Saint-Georges; Centre hospitalier de Versailles, Le Chesnay; and Hôpital Louis Mourier (AP-HP), Colombes. The inclusion potential of each center was determined from the number of deliveries per year at the center concerned.

### Inclusion criteria

The women included in this study will have the following characteristics:

- between the ages of 18 and 44 years, covered by the social security system and presenting with trophoblastic intrauterine retention following incomplete spontaneous abortion after the first trimester (<14 weeks of amenorrhea)
- the pregnancy concerned should correspond to a planned/wanted baby
- diagnosis of intrauterine retention by transvaginal pelvic ultrasound showing a heterogeneous intrauterine mass or an intrauterine sac over 15 mm thick, with or without endometritis
- selected by the medical team for surgical management on the basis of local management protocols
- written consent provided.

### Exclusion criteria

The exclusion criteria are as follows:

- known uterine malformations
- history of surgical treatment for intrauterine retention
- intrauterine retention of material over 50 mm thick, diagnosed by transvaginal pelvic ultrasound
- emergency hemostatic therapy to treat heavy vaginal bleeding (hemorrhagic miscarriage)
- presence of an intrauterine device
- ongoing pregnancy
- extra uterine pregnancy
- trophoblastic retention following an abortion;
- pregnancy obtained by medically assisted procreation.

### Interventions

**Fig. 1:**
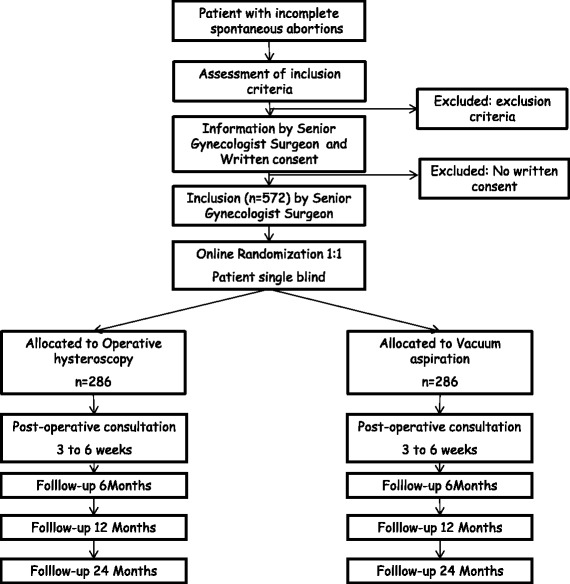
Patient flow chart

The design of the study is summarized in a schematic diagram and a patient flow chart (Fig. 1 and Table 1).

**Table 1:** Time schedule of enrollment

Timetable	Inclusion	Intervention	Postoperative consultation 3 to 8 weeks	Follow up 6 months	Follow up	
					1 year	2 years
Information notice	X					
Written consent	X					
Gynecological examination	X		X			
Transvaginal ultrasound	X					
Randomization	X	X				
Intervention		X				
Recording of adverse effects		X	X	X	X	X
Questionnaire for pregnancy counts				X	X	X

The interventions to be evaluated are operative hysteroscopy (arm A) and vacuum aspiration (arm B) for the management of incomplete spontaneous abortions. These procedures are routinely performed in the participating centers, for various indications, by the local investigators. For both surgical procedures, surgical antibiotic prophylaxis, misoprostol to dilate the cervix, and anti-adhesion barrier gels will be used, according to the routine procedures of each center. In cases of intrauterine retention complicated by endometritis, antibiotic treatment for 48 hours (penicillin, metronidazole, fluoroquinolones or a combination of these antibiotics) will be administered according to the standard practice of each center. The evacuated retention product will be sent for pathological examination. Rhesus-negative women will receive prophylaxis to prevent Rh alloimmunization.

#### Arm A: operative hysteroscopy

Operative hysteroscopy is a routine surgical procedure for the management of intrauterine disease in obstetrics and gynecology departments. The procedure will be performed by a gynecological surgeon, under general or local anesthesia, according to the standard practice of the center concerned, with the patient in the gynecological position. Antibiotic prophylaxis may be administered, according to the standard practice of the center. The cervix is grasped with pozzi forceps and dilated, with up to a size-9 Hegar dilator if necessary, to facilitate insertion of the hysteroscope. The equipment available at the center will be used for operative hysteroscopy. The uterine cavity will be distended with saline or glycine, depending on the polarity of the high-frequency generator required for the resection system (monopolar or bipolar), with a maximum irrigation pressure of 110 mm Hg. The use of energy is usually unnecessary and saline will be the preferred method. Before the operation, the appearance of the uterine cavity will be recorded for diagnostic purposes. The retained conception product will be resected from top to bottom with the surgical resector, without electric power, this being the method recognized as best protecting the endometrium in previous studies [7, 8]. The use of forceps or curettes to facilitate the removal of material is permitted. An electrical current will be used only as a last resort, in cases in which the retained conception product cannot be removed without it. If active bleeding occurs after surgery, elective coagulation via the hysteroscope may be carried out to stop intrauterine bleeding. The distension medium will be measured and examined on entry and exit.

#### Arm B: vacuum aspiration

Vacuum aspiration will be carried out by a gynecological surgeon, according to the standard protocol of the center. A flexible or rigid vacurette may be used. Antibiotic prophylaxis, the diameter of the vacurette used, the extent of cervical dilatation necessary and the use of intraoperative ultrasound guidance will be left to the discretion of the operator and the standard practice of the center. In most centers, the cervix is dilated with a size-9 Hegar dilator for vacuum aspiration. In some centers, the expansion is minimized (in practice, Hegar dilator size 7) to limit the risk of subsequent preterm birth. However, published studies have not reported any benefits of moderate over maximal cervical dilation. The participating centers will therefore be free to carry out cervical dilation according to their standard practices and to use the size and type of vacurette of their choice. In arm A, the use of forceps or curettes to facilitate the removal of material is permitted during the procedure. The scoop of the curette is usually examined to ensure that the uterus is empty at the end of curettage and this practice will be authorized in this trial.

### Examination on inclusion

The initial assessment will include a complete gynecological examination. The patient’s hemodynamic variables (heart rate, blood pressure, temperature) will be recorded and signs of shock sought, to identify women susceptible to hemorrhagic shock in cases of heavy bleeding (exclusion criterion). The last menstrual period of the patient and/or the exact start date of pregnancy in cases in which the pregnancy was dated by early pelvic ultrasound will be recorded. The pelvic examination should include (1) a speculum examination to search for bleeding and to evaluate its extent and source and (2) a vaginal examination to assess pain originating in the uterus or elsewhere.

Incomplete spontaneous abortion will be diagnosed by transvaginal ultrasound performed according to a previously published standard technique for pelvic ultrasound [13]. It must include the median sagittal section of the uterus passing through the endocervical cavity and a section centered on the uterine cavity and passing through the thickest part of the retained conception product, the dimensions of which will be measured. Intrauterine retention will be diagnosed on the basis of the visualization of a heterogeneous intrauterine mass or an intrauterine sac over 15 mm thick.

An ultrasound scan showing a visible intrauterine pregnancy before the expulsion will be required to confirm the intrauterine nature of the pregnancy and to exclude extra-uterine pregnancies. If the intrauterine nature of the pregnancy or its stage cannot be confirmed, β human chorionic gonadotropin (βhCG) levels will be analyzed over 48 hours. A 50 % decrease in βhCG levels over 48 hours will be considered to confirm that the interrupted pregnancy was indeed intrauterine [14]. The intrauterine nature of the pregnancy will be further confirmed by histological analysis of the retained conception product.

Women experiencing hyperthermia or pelvic pain will be examined for endometritis, which may occur as a complication of incomplete miscarriage. Endometritis will be diagnosed according to the criteria in the 2012 guidelines for clinical practice from the French National College of Obstetricians-Gynecologists [15]: the presence of pain following uterine mobilization associated with hyperthermia or high C-reactive protein (CRP) levels, a positive vaginal swab or signs of endometritis on endometrial biopsy. If the blood group of the patient is unknown, rhesus status will be determined and prophylaxis for Rh alloimmunization will be initiated if the patient is Rh-negative.

During this visit, the patient will complete a self-administered questionnaire on her medical history and the lost pregnancy. Details of the protocol will be provided to the patient through a written notice and oral explanations. Signed consent forms will be collected from patients agreeing to participate in the study prior to enrollment, by a senior gynecologist acting as a local investigator in the trial. Informed consent form is shown in Additional file 4.

### Randomization

After the patient has been informed, written informed consent obtained and the criteria for inclusion and exclusion have been checked, a single-blind randomization with a 1:1 ratio will be performed electronically with a secure internet platform, on arrival in the operating room.

The patient will not be informed of the procedure that she has undergone. In addition, the operating rooms will be systematically prepared for hysteroscopy, so that the patient cannot guess the nature of the procedure performed. The intervention will be unblinded if patients experience adverse effects during the study period.

### Intrahospital management

During their stay in hospital, the patients will be managed according to the standard practice of the center. A standardized surgical report will be given to patients included in the study, with no mention of the type of surgical procedure. The patient will then receive a detailed surgical report at the end of her participation in the trial.

### Postoperative consultation

Patients will attend a postoperative consultation within three to eight weeks of the intervention, to check for the absence of complications following the procedure and exposure to pregnancy. Postoperative management, in terms of monitoring, is identical for curettage and hysteroscopy; it should therefore be possible to maintain the single-blind design. If a procedure or special assessment needs to be carried out according to the standard practice of the center (assessment of spontaneous abortion, pelvic ultrasound, diagnostic hysteroscopy) it will be performed without compromising the single-blinded nature of the study. According to amendment of the protocol v2.1 dated 6 May 2015, patients may also be contacted by phone if they do not attend the postoperative consultation, to check for the absence of adverse effects.

### Follow up

After their initial postoperative consultation, patients will attend follow up 6 months, one year and 2 years after surgical treatment or until the successful completion of a subsequent pregnancy. Thus, the length of participation of each patient starts from their inclusion in the study, lasting until subsequent childbirth, or a maximum of 2 years. Each patient will be contacted by telephone. In the absence of a reply, an email and/or a letter will be sent. Subsequent pregnancies, surgical procedures and gynecological findings, such as the diagnosis of abnormal intrauterine pathology (by diagnostic hysteroscopy, hysterosonography, hysterography or pelvic ultrasound) will be recorded. Women will be asked about their exposure to pregnancy (sexual intercourse without contraception), and their use of medical procedures for procreation. The dates of the beginning and the end of any subsequent pregnancies (miscarriage, abortion, ectopic pregnancy, childbirth) will be recorded. The blinding of the study will be assessed during interviews with the patients during follow up. Patients may reply that (1) they do not know which arm they were assigned to, (ii) they think they underwent hysteroscopy, or (iii) they think they underwent vacuum aspiration.

The primary endpoint of the trial is a pregnancy of at least 22 weeks duration in the 2 years of follow up. The progress of the pregnancy will be evaluated according to standard procedures. These data will be collected from a standardized report of the pregnancy and birth (see Additional file 5). In cases of delivery at the same hospital or of additional examinations or surgical procedures, medical records may be used to complete the information.

### Sample size

The mean live birth rate at 2 years after intrauterine retention following miscarriage is 60 % [1]. To demonstrate that the use of hysteroscopy increases this rate by 20 %, i.e., to 72 % with a power of 80 % and α = 0.05 (two-tailed), the number of patients required for this study is 520 (260 per arm) [16, 17]. Assuming that 10 % of patients in each group will be lost to follow up, we plan to include 286 patients in each arm, or 572 patients in total.

### Handling of missing data

Participants lost to follow up will be taken into account on the basis of their last known status and several reminders will be sent. We will check that the participants lost to follow up are equally distributed between the two arms of the trial, which is the usual procedure to deal with such participants in this type of trial. An explanatory calculation may be performed to classify these participants as failures (no pregnancy of more than 22 weeks of gestation within 2 years). Methods based on multiple imputation may also be used.

### Statistical analysis

The intention-to-treat principle will be used during statistical analysis; thus, the analysis will be performed on the final list of all randomized patients included in the trial. The main outcome measure will be a comparison between the two arms of the proportion of patients with a pregnancy lasting for more than 22 weeks gestation in the 2 years of follow up, based on the chi-squared test. Multiple pregnancies (i.e., twin pregnancy outcome: incidence of miscarriage and ectopic pregnancy during the 2-year follow-up period or more) will be counted as a single pregnancy lasting for at least 22 weeks of gestation in this comparison.

A secondary analysis will be performed by the Kaplan-Meier method [18]. The event considered for time-to-event analyses will be the occurrence of a pregnancy lasting for at least 22 weeks, with measured exposure to pregnancy (desire to become pregnant, with no use of contraception). In the time-to-event analyses, patients with a successful pregnancy will be counted as successful outcomes and will then be censored. No other pregnancy in the 2 years of follow up will be taken into account for these patients. By contrast, the event of interest will not be considered to have occurred in patients experiencing a loss of the pregnancy before 22 weeks of gestation and their follow up will thus continue for 2 years or until a pregnancy lasting at least 22 weeks is achieved. In this analysis, the log-rank test will be used to compare conception rates between the two randomization groups [19]. Cox proportional hazards regression [20] will be used to take into account possible confounding factors. We also will estimate the probability of conception after treatment, by measuring monthly fertility and its 95 % confidence interval from the number of spontaneous conceptions per person per month of follow up. Secondary endpoints (incidence of miscarriage and ectopic pregnancy in the 2-year follow-up period; surgical complications according to the Clavien-Dindo classification; secondary surgical intervention for the management of intrauterine retention after miscarriage) will be analyzed and compared between the two arms by chi-squared test. All tests will be two-tailed. Interim analyses are not planned. Stata version 13 software (Stata Corp., College Station, TX, USA) will be used for all statistical analyses.

### Data collection and management

Data for all phases of the study will be collected in a stepwise manner using the secure online internet platform, Cleanweb (https://cleanweb.aphp.fr/Ctms-02/portal/login). Personal information relating to the participants enrolled will be known only to the local investigators. The first letter of the given name and the first letter of the last name, together with the month and year of birth, will be collected for each patient, to protect confidentiality in the database.

As neither the investigators nor the sponsor have any competing interests and the study does not focus on the use of drugs, no data monitoring committee is required. Data quality will be monitored by visits to the investigating centers, with a frequency corresponding to the planned follow up of patients in the protocol, inclusions at the various centers and the level of risk attributed to the protocol.

During subsequent visits, the observations will be reviewed as the study progresses, by clinical research assistants, who will be responsible for checking that the data are correctly entered and for validating the data. The principal investigator at each center and the other investigators responsible for including or following up subjects participating in the study will agree to visits from representatives of the promoter, nominated by Assistance Publique-Hôpitaux de Paris (AP-HP), at regular intervals. During these site visits, in accordance with good clinical practice, the following aspects will be audited:

- respect for the research protocols and the procedures defined in it
- examination of the source documents, comparing them with the data reported in the online observation notebook, to assure the quality of the results obtained: exactness, missing data, data consistency.

### Responsibility

AP-HP is the promoter for this study. In accordance with biomedical research law, AP-HP has taken out an insurance policy with GERLING KonZern for the entire duration of the study, covering its own civil responsibility and that of all those involved in the study (doctors and staff involved in carrying out the research; law no. 2004–806, Art. L.1121-10 of the CSP).

### Publication and ownership of the results

AP-HP is the owner of all the data obtained, and no use or transmission of these data to a third party is permitted without prior agreement. The individuals who made a genuine contribution to the development and running of the protocol and to the writing up of the results will be the first authors on any resulting publications. The principal investigator and methodologists will be authors on the principal publication. The positions of the intermediate authors will depend on the number of patients included and participation in the writing of the manuscript (including participation in study design, data analysis and the writing and reviewing of the manuscript).

## Discussion

This study is a single-blind randomized controlled trial. The single-blind design was chosen so as to restrict any placebo effect on the occurrence of pregnancy. A double-blind design was not possible because the two arms of the study correspond to surgical interventions. Blinding for the patient will be assessed during follow up, to determine the efficacy of the patient blinding procedure.

The validity and reliability of data collection procedures used in the trial could be called into question as follow up is planned to involve telephone conversations or questionnaires sent by email or post during a 2-year period, rather than a review of medical records. However, patients often move house just before or after giving birth. The patient might then give birth at another hospital and would be lost to follow up. In cases of delivery at the same hospital, medical records could be used to complete the information. Moreover, we took a loss-to-follow-up rate of 10 % into account when determining sample size.

If our study provides evidence that the use of operative hysteroscopy can increase the number of live births in the two-year period following incomplete spontaneous abortion without increasing the incidence of morbidity and perioperative and postoperative complications, then there will be a change in the standard surgical treatment of this condition. This study may therefore have a major impact on the surgical management of incomplete spontaneous abortion.

## Trial status

The HY-PER trial is currently in the recruitment phase.

## Additional files

Additional file 1:
**Items from the World Health Organization Trial Registration Data Set for the Operative hysteroscopy versus vacuum aspiration for incomplete spontaneous abortion (HY-PER) trial.** (DOC 45 kb) Additional file 2:
**The TIDieR (Template for Intervention Description and Replication) Checklist for the Operative hysteroscopy versus vacuum aspiration for incomplete spontaneous abortion (HY-PER) trial.** (PDF 391 kb) Additional file 3:
**SPIRIT (Standard Protocol Items: Recommendations for Interventional Trials) 2013 Checklist: Recommended items to address in a clinical trial protocol and related documents for the Operative hysteroscopy versus vacuum aspiration for incomplete spontaneous abortion (HY-PER) trial.** (PDF 129 kb) Additional file 4:
**Informed consent for the Operative hysteroscopy versus vacuum aspiration for incomplete spontaneous abortion (HY-PER) trial.** (PDF 285 kb) Additional file 5:
**Follow up questionnaire of the Operative hysteroscopy versus vacuum aspiration for incomplete spontaneous abortion (HY-PER) trial.** (PDF 231 kb)

## Abbreviations

AP-HP: Assistance Publique–Hôpitaux de Paris
βHCG: β Human chorionic gonadotropin
CPP: Person Protection Committee
CRP: C- Reactive protein
HY-PER: Operative hysteroscopy versus vacuum aspiration for incomplete spontaneous abortion
Rh: rhesus
