# Supplementary material for: Operative hysteroscopy versus vacuum aspiration for incomplete spontaneous abortion (HY-PER): study protocol for a randomized controlled trial
Source: Trials. 2015 Aug 19;16:363. doi: 10.1186/s13063-015-0900-1 (PMC4539935; doi:10.1186/s13063-015-0900-1)
Supplement: Additional file 1: — Items from the World Health Organization Trial Registration Data Set for the Operative hysteroscopy versus vacuum aspiration for incomplete spontaneous abortion (HY-PER) trial. (DOC 45 kb) [file 13063_2015_900_MOESM1_ESM.doc]

**Appendix with all items from the World Health Organization Trial Registration Data Set for the HY-PER trial.**

| **Data category** | **Information** |
| --- | --- |
| Primary registry and trial identification number | ClinicalTrials.gov Identifier: NCT02201732; |
| Date of registration in primary registry | 17 July 2014. |
| Secondary identifying numbers | P130920, 2014-A00340-47 |
| Source(s) of monetary or material support | Assistance Publique - Hôpitaux de Paris |
| Primary sponsor | Assistance Publique - Hôpitaux de Paris |
| Secondary sponsor(s) | - |
| Contact for public queries | Dr Cyrille Huchon, MD, PhD , (33) 1 39 27 45 76, cyrillehuchon@yahoo.fr |
| Contact for scientific queries | Dr Cyrille Huchon, MD, PhD , (33) 1 39 27 45 76, cyrillehuchon@yahoo.fr |
| Public title | Effectiveness of Hysteroscopy in the Treatment of Intrauterine Trophoblastic Retentions |
| Scientific title | Assessment of the Effectiveness of Hysteroscopy in the Treatment of Intrauterine Trophoblastic Retentions (HY-PER) |
| Countries of recruitment | France |
| Health condition(s) or problem(s) studied | Incomplete Miscarriage |
| Intervention(s) | Procedure: Hysteroscopy  Procedure: Curettage |
| Key inclusion and exclusion criteria | Inclusion Criteria:   - Major, under 45 years with intrauterine trophoblastic retention after incomplete miscarriage in the first trimester (termination of pregnancy < 14 WA), - With desire to become pregnant; - Intrauterine trophoblastic retention diagnosed by transvaginal pelvic ultrasound finding a intrauterine heterogeneous image or intrauterine gestational sac more than 15 mm in thickness, with or without endometritis; - Decision taken by the healthcare team to manage the incomplete miscarriage surgically; - Patient covered by or affiliated to a health insurance - Written informed consent   Exclusion criteria:  Patients will not be included in the protocol if:   - They have a known uterine malformation; - They have undergone surgical treatment for the current intrauterine retention; - The intrauterine retention diagnosed by transvaginal pelvic ultrasound is more than 50 mm thick; - Emergency hemostatic treatment is required for heavy vaginal bleeding (miscarriage hemorrhage) ; - If the patient has an intrauterine device; - The patient has a progressive pregnancy; - The patient has an ectopic pregnancy; - The patient has trophoblastic retention following an elective abortion; - The pregnancy was achieved by medically assisted procreation |
| Study type | Interventional Allocation: randomized  Endpoint classification: efficacy study Intervention model: parallel assignment Masking: single-blind (subject) Primary purpose: treatment Phase III |
| Date of first enrolment | November 2014 |
| Target sample size | 572 |
| Recruitment status | Recruiting |
| Primary outcome(s) | Progressive intrauterine pregnancy > 22 WG [Timeframe: until 2 years post surgery] [Designated as safety issue: no] |
| Key secondary outcomes | Time to conception [Timeframe: until 2 years post surgery] [Designated as safety issue: no]  Surgical complication rate [Timeframe: until 2 years post surgery] [Designated as safety issue: No] Clavien-Dindo classification  Further surgery [Timeframe: until 2 years post surgery] [Designated as safety issue: no] |
