# Supplementary material for: Operative hysteroscopy versus vacuum aspiration for incomplete spontaneous abortion (HY-PER): study protocol for a randomized controlled trial
Source: Trials. 2015 Aug 19;16:363. doi: 10.1186/s13063-015-0900-1 (PMC4539935; doi:10.1186/s13063-015-0900-1)
Supplement: Additional file 4: — Informed consent for the Operative hysteroscopy versus vacuum aspiration for incomplete spontaneous abortion (HY-PER) trial. (PDF 285 kb) [file 13063_2015_900_MOESM4_ESM.pdf]

Titre complet de recherche :
« Evaluation de l'efficacité de l'hystéroscopie dans la prise en charge des rétention trophoblastiques intra utérines ou étude HY-PER »

Cette recherche est organisée par l'Assistance Publique - Hôpitaux de Paris
Département de la Recherche Clinique et du Développement
1 avenue Claude Vellefaux
75010 Paris

NOTE D'INFORMATION

Madame,

Vous avez fait un avortement spontané incomplet et il est prévu une intervention chirurgicale pour éliminer la rétention utérine**.** Plusieurs techniques sont disponibles et le Docteur…………………………………. (nom, prénom), exerçant à l'hôpital ……………………………………………., vous propose de participer à une recherche comparant deux techniques chirurgicales qui seront comparées.
Il est important de lire attentivement cette note avant de prendre votre décision ; n'hésitez pas à lui demander des explications.
Si vous décidez de participer à cette recherche, un consentement écrit vous sera demandé.

1) Quel est le but de cette recherche?

Cette recherche porte sur le traitement chirurgical des avortements spontanés incomplets chez les patientes désireuses de grossesse. Son but est de déterminer la meilleure technique chirurgicale pour l'obtention d'une grossesse à son terme après une fausse couche incomplète.
Pour répondre à la question posée dans cette recherche, il est prévu d'inclure *572* femmes présentant une rétention de produit de grossesse à l'intérieur de l'utérus pour lesquelles a été décidé un traitement chirurgical. Plusieurs établissements de soins, situés en France, participeront à cette recherche.

2) En quoi consiste la recherche ?

L'objectif de cette recherche est de comparer le curetage aspiratif, traitement de référence actuel des retentions trophoblastiques à l'hystéroscopie opératoire qui permet une visualisation directe et une évacuation élective et qui laisse espérer une augmentation de la fertilité par rapport au curetage aspiratif. Vous serez affectée par tirage au sort, (seule méthode permettant d'avoir une évaluation objective) dans un de ces 2 groupes suivants: soit du traitement chirurgical par hystéroscopie, soit du traitement chirurgical par curetage afin d'évacuer la rétention intra-utérine que vous présentez. Ces 2 techniques sont habituellement utilisées dans les hôpitaux participant à cette recherche
Le curetage, actuellement technique de référence, est une procédure chirurgicale réalisée par votre médecin permettant d'aspirer les rétentions à l'intérieur de l'utérus après avoir introduit à l'intérieur de celui-ci une canule. L'hystéroscopie opératoire consiste à mettre une caméra à l'intérieur de l'utérus et réaliser une évacuation sous contrôle de la vue du contenu utérin à l'aide d'une anse permettant d'attraper sélectivement ce contenu.

3) Déroulement de la recherche ?

La recherche durera *4* années et votre participation sera au maximum de *24* mois. Après la signature de votre consentement, lors de la première visite (visite d'inclusion) et après randomisation (tirage au sort de la méthode chirurgicale utilisée pour vous) l'intervention chirurgicale sera programmée Puis, vous aurez une visite postopératoire 3 à 6 semaines après l'opération où votre chirurgien s'assurera que tout s'est bien passé depuis l'intervention. Dans le cas où vous ne serez pas revue en consultation, vous serez alors contactée par téléphone afin de s'assurer qu'il n'y a pas eu de complication. Vous serez ensuite contactée 6 mois après l'intervention par entretien téléphonique ou nous vous poserons des questions sur votre état

de santé et votre projet de grossesse ou votre grossesse si elle était en cours. Le but de ce questionnaire est de s'assurer de votre état de santé et l'absence d'évènements indésirables suite à l'intervention ou survenus au décours du début d'une nouvelle grossesse.. Ensuite, vous serez contactée un an après, de préférence par téléphone pour compléter de nouveau ce questionnaire et de vous interroger sur la survenue d'une grossesse. Si une grossesse n'était pas survenue au bout d'un an, nous vous recontacterons deux ans après l'intervention chirurgicale de la même manière.

Nous pourrons également vous contacter par courrier où courriel, si nous n'arrivons pas à vous joindre par téléphone ou si vous le préférez.

En cas de non-participation à la recherche, le traitement chirurgical qui vous sera proposé sera celui décidé par votre gynécologue.

Cette recherche ne modifie en rien votre prise en charge habituelle
Aucun suivi ne sera nécessaire à la fin de l'étude. Votre participation s'arrêtera à l'obtention d'une grossesse évolutive ou à l'issue des deux ans maximaux de suivi si vous n'avez pas été enceinte durant cette période.
Votre médecin pourra décider à tout moment de l'arrêt de votre participation ; il vous en expliquera les raisons.

4) Quels sont les bénéfices et les contraintes liés à votre participation ?

En participant à cette recherche, vous bénéficierez d'un suivi médical étroit et spécifique pour lequel aucun frais supplémentaire ne vous sera demandé. Par ailleurs, vous contribuerez à une meilleure connaissance de la prise en charge chirurgicale des avortements spontanés incomplets.

Si vous acceptez de participer, vous devrez respecter les points suivants :

- Venir au rendez-vous post-opératoire. En cas d'impossibilité, nous vous remercions de contacter votre médecin le plus rapidement possible.
- Répondre à un ou deux questionnaires sur votre fertilité dans les deux ans suivant votre prise en charge.
- Etre affiliée à un régime de sécurité sociale ou être bénéficiaire d'un tel régime.

5) Quels sont les traitements autorisés et non autorisés ?

Tous les traitements sont autorisés pendant la période de l'étude en dehors de la période pré-anesthésique où la prise d'un traitement doit être autorisée par l'anesthésiste.

6) Quels sont les risques prévisibles de la recherche?

Les risques prévisibles de la recherche sont ceux de votre prise en charge chirurgicale habituelle dans le cadre de vos soins. Outre les éventuels risques anesthésiques, les procédures chirurgicales visant à obtenir l'évacuation de l'utérus peuvent être source de rares complications, à savoir l'échec de l'intervention, une perforation de l'utérus ou encore une hémorragie génitale pouvant nécessiter la mise en œuvre de mesures spécifiques telles que transfusion de produit sanguin ou procédure chirurgicale intra-abdominales. A distance de l'intervention, des adhérences à l'intérieur de l'utérus, appelées synéchies, sont une des complications fréquentes connues des curetages.

7) Quelles sont les modalités de prise en charge médicale à la fin de votre participation?

Cette recherche ne modifie en rien votre prise en charge habituelle
Aucun suivi ne sera nécessaire à la fin de l'étude. Votre participation s'arrêtera à l'obtention d'une grossesse évolutive ou à l'issue des deux ans maximaux de suivi si vous n'avez pas été enceinte durant cette période.
Votre médecin pourra décider à tout moment de l'arrêt de votre participation ; il vous en expliquera les raisons.

8) Comment cette recherche est-elle encadrée ?

L'AP-HP a souscrit une assurance (N° d'adhésion : 0100518814033-130046-10998) garantissant sa responsabilité civile et celle de tout intervenant auprès de la compagnie HDI–GERLING par l'intermédiaire de BIOMEDICINSURE dont l'adresse est Parc d'Innovation Bretagne Sud C.P.142 56038 Vannes Cedex.

L'AP-HP a pris toutes les dispositions prévues par la loi relative à la protection des personnes se prêtant à des recherches biomédicales, loi Huriet (n° 88-1138) du 20 décembre 1988 modifiée par la loi de santé publique (n° 2004-806) du 9 août 2004.

L'AP-HP a obtenu l'avis favorable du Comité de Protection des Personnes CPP Ile de France IX Poissy Saint germain pour cette recherche le 16/06/2014 et une autorisation de l'Agence Nationale de Sécurité du Médicament et des produits de santé (ANSM).

RECUEIL ET TRAITEMENT DES DONNEES:

Dans le cadre de la recherche biomédicale à laquelle l'Assistance publique - hôpitaux de Paris vous propose de participer, vos données individuelles nécessaires à l'étude rassemblées sous forme anonyme, sont transmises au promoteur pour être traitées par informatique afin d'analyser les résultats de la recherche au regard de l'objectif qui vous a été présenté.

Elles peuvent aussi être transmises aux Autorités de Santé Françaises

D'autre part, le représentant du promoteur ou celui des Autorités de Santé, tenu au secret professionnel, peut avoir accès à votre dossier pour contrôle de conformité.

Conformément aux dispositions de la loi relative à l'informatique, aux fichiers et aux libertés, le fichier a reçu l'autorisation de la CNIL et vous disposez d'un droit d'accès et de rectification que vous pouvez exercer auprès du médecin qui vous suit dans le cadre de la recherche et qui, seul connaît votre identité.

Vous disposez également d'un droit d'opposition à la transmission des données couvertes par le secret professionnel susceptibles d'être utilisées et traitées dans le cadre de cette recherche.

Vous pouvez également accéder directement ou par l'intermédiaire d'un médecin de votre choix à l'ensemble de vos données médicales en application des dispositions de l'article L1111-7 du code de la santé publique

AUTRES ASPECTS REGLEMENTAIRES

Si vous le souhaitez, vous pourrez être informée par le médecin investigateur ou par la sage-femme des résultats globaux de cette recherche.

Votre participation à cette recherche est libre. De plus, vous pouvez exercer à tout moment votre droit de retrait de cette recherche. Celui-ci qui n'aura pas de conséquence sur votre prise en charge ou sur votre relation avec les équipes soignantes,

Lorsque vous aurez lu cette note d'information et obtenu les réponses aux questions que vous vous posez en interrogeant la sage-femme ou le médecin, il vous sera proposé, si vous êtes d'accord, de donner votre consentement écrit en signant le formulaire préparé à cet effet

FORMULAIRE DE CONSENTEMENT

Je soussigné(e), Mme, (nom, prénom)……………………………………………………...
accepte librement de participer à la recherche intitulée

« EVALUTION DE L'EFFICACITE DE L'HYSTEROSCOPIE DANS LA PRISE EN CHARGE DES RETENTIONS TROPHOBLASTIQUES INTRA-UTERINES (HY-PER)»
organisée par l'Assistance Publique - Hôpitaux de Paris et qui m'est proposée par le Docteur (nom, prénom, téléphone)………………………………………………………………………….…, médecin dans cette recherche.

- J'ai pris connaissance de la note d'information version N°2 du 06 mai 2015 (4 pages) m'expliquant l'objectif de cette recherche, la façon dont elle va être réalisée et ce que ma participation va impliquer,
- je conserverai un exemplaire de la note d'information et du consentement,
- j'ai reçu des réponses adaptées à toutes mes questions,
- j'ai disposé d'un temps suffisant pour prendre ma décision,
- j'ai compris que ma participation est libre et que je pourrai interrompre ma participation à tout moment, sans encourir la moindre responsabilité et préjudice pour la qualité des soins qui me seront prodigués. J'indiquerai alors au médecin qui me suit, si je souhaite ou non que les données recueillies, jusqu'au moment de ma décision, soient utilisées,
- Je suis consciente que ma participation pourra aussi être interrompue par le médecin si besoin,
- avant de participer à cette recherche, j'ai bénéficié d'un examen médical adapté à la recherche, dont les résultats m'ont été communiqués,
- j'ai compris que pour pouvoir participer à cette recherche je dois être affiliée à un régime de sécurité sociale ou bénéficiaire d'un tel régime. Je confirme que c'est le cas,
- j'ai bien été informée que ma participation à cette recherche durera 24 mois au maximum
  mon consentement ne décharge en rien le médecin qui me suit dans le cadre de la recherche ni l'AP-HP de l'ensemble de leurs responsabilités et je conserve tous mes droits garantis par la loi.
- j'ai bien été informée que je serai contactée à 6 mois, 1 an et 2 ans. Ce contact sera fait selon ma préférence soit
    - par téléphone (tél :……………………………..)
    - ou par mail ou courrier (mail :………………………………..)

Signature de la personne participant à la recherche	Signature du médecin
Nom Prénom :	Nom Prénom :
Date :	Date :
Signature :	Signature :

Ce document est à réaliser en 3 exemplaires, dont l'original doit être conservé 15 ans par l'investigateur, le deuxième remis à la personne donnant son consentement et le troisième transmis à l'AP-HP sous enveloppe scellée à la fin de la recherche.
