# Supplementary material for: Operative hysteroscopy versus vacuum aspiration for incomplete spontaneous abortion (HY-PER): study protocol for a randomized controlled trial
Source: Trials. 2015 Aug 19;16:363. doi: 10.1186/s13063-015-0900-1 (PMC4539935; doi:10.1186/s13063-015-0900-1)
Supplement: Additional file 5: — Follow up questionnaire of the Operative hysteroscopy versus vacuum aspiration for incomplete spontaneous abortion (HY-PER) trial. (PDF 231 kb) [file 13063_2015_900_MOESM5_ESM.pdf]

ETUDE *Hy-Per*

Chère Mademoiselle, Chère Madame,

Vous avez accepté de participer au protocole HYPER comparant l'efficacité de l'hystéroscopie au curetage dans le traitement chirurgical des avortements spontanés incomplets.

Nous vous remercions d'accepter de répondre à cet auto-questionnaire.

Les renseignements que vous nous fournissez sont confidentiels et feront l'objet d'un traitement statistique anonyme dans le cadre de l'étude HYPER.
Avec nos remerciements pour votre collaboration

Nom : …………………………

Prénom : ……………………..

A quelle date répondez-vous à ce questionnaire ?

……. /……/…201…………….

1ere Partie : Suivi de l'acte chirurgical :

Avez-vous eu une complication depuis votre traitement chirurgical ?
Non ☐
Oui ☐

Si **Oui**, laquelle : …………………

Avez-vous du être hospitalisée pour cette complication ?
Non ☐
Oui ☐

Si oui,

A quelle date : …………………….

Dans quel hôpital ou clinique : ……………………

Avez-vous eu une nouvelle intervention chirurgicale pour cette complication ?

Non ☐

Oui ☐

Si oui :

A quelle date : …………….........

Pour quel motif :………………….

Dans quel hôpital ou clinique : ……………………

Quel est le nom du médecin : ……………………..

Pouvez-vous préciser l'intervention :

☐ - Hystéroscopieopératoire
☐ - Curetage
☐ - Coelioscopie
☐ - Laparotomie
☐ - Embolisation en radiologie interventionnelle

Avez-vous eu une hystéroscopie diagnostique depuis votre intervention ?

Non ☐

Oui ☐

Si **oui**, était-elle normale ?

Non ☐

Oui ☐

Si **non**, quelle anomalie était-elle présente ? ………………………

Savez-vous actuellement dans quel bras de l'étude vous étiez, c'est-à-dire quel traitement vous avez reçu, entre l'hystéroscopie et le curetage aspiratif,lors de votre prise en charge initiale ?

☐ Je ne sais pas

☐ Je pense avoir reçu l'hystéroscopie

☐ Je pense avoir reçu le curetage aspiratif

<u>2eme Partie : Désir de grossesse.</u>

Etes-vous actuellement enceinte ?

Non ☐
Oui ☐

Si Oui :
Quelle est la date estimée du début de grossesse ? ……. /……/…201…………….

Elle a été estimée par

☐ La date de vos dernières règles

☐ Echographie

Si **non** :

Essayez-vous actuellement- d'être enceinte ?

Non ☐
Oui ☐

Si Oui :
Depuis quelle date ?……. /……/…201…………….

Avez-vous actuellement des rapports sexuels dans le but d'obtenir une grossesse ?

Non ☐
Oui ☐

Etes-vous actuellement en procédure d'Assistance Médicale à la Procréation ?

Non ☐
Oui ☐

Si **oui**,

Depuis quelle date……. /……/…201…………….

Quelle méthode est utilisée ?:

☐Stimulation simple

☐Insémination intra utérine (IIU)

☐Fécondation in-vitro (FIV)

☐Autre, précisez : ……………….

Utilisez-vous actuellement une **méthode contraceptive** ?

Non ☐
Oui ☐

Si **OUI** :

Veuillez préciser le jour de début de contraception
……. /……/…201……………

Quelle méthode utilisez-vous ?
☐ Une pilule
Si vous UTILISEZ ACTUELLEMENT une pilule contraceptive, précisez le **nom du produit** :...

☐ Un stérilet
Si vous UTILISEZ ACTUELLEMENT un stérilet, précisez le **nom du produit** : ……..
....

☐ Un implant sous cutané

☐ Autre : détaillez :

Avez-vous été enceinte depuis l'intervention chirurgicale ?

Non ☐
Oui ☐

Si **Oui** :
Quelle a été la date estimée du début de grossesse ?……. /……/…201……………

Elle a été estimée

☐Par la date de vos dernières règles

☐Par échographie

Quelle a été la date de la fin de cette grossesse ?……. /……/…201……………

S'agissait-il d'une Grossesse

☐Simple

☐Gémellaire

☐3 ou plus

L'issue de cette grossesse était :

☐Accouchement

☐FCS (fausse couche spontanée précoce ou tardive)

☐GEU (grossesse extra utérine)

☐IVG (interruption volontaire de grossesse)

Autre :

En cas d'accouchementpourriez-vous préciser

L'hôpital ou la clinique ou vous avez accouché : ……………

La date de l'accouchement : ………………….

Le nombre d'enfant : …………………………..

En cas d'accouchementpourriez-vous préciser la méthode

☐Accouchement par les voies naturelles

☐Césarienne

Toujours en cas d'accouchement, avez-vous eu des pathologies pendant la grossesse ?

☐**Non**

☐**Oui**

Si **Oui** :

☐Menace d'accouchement prématuré

☐Retard de croissance intra-utérin

☐Hémorragie de la délivrance

☐Autre : détaillez

Avez-vous eu un problème pour l'évacuation du placenta après l'accouchement ?

☐**Non**

☐**Oui**

Si **Oui**, précisez : …………………….

En vous remerciant.

L'équipe de l'étude HYPER
